# Complex-Amplitude-Modulation Vectorial Excitation Beam for High-Resolution Observation of Deep Regions in Two-Photon Microscopy

**DOI:** 10.3389/fnins.2022.880178

**Published:** 2022-04-19

**Authors:** Naoya Matsumoto, Koyo Watanabe, Alu Konno, Takashi Inoue, Shigetoshi Okazaki

**Affiliations:** ^1^Central Research Laboratory, Hamamatsu Photonics K.K., Hamamatsu, Japan; ^2^Hamamatsu BioPhotonics Innovation Chair, Institute for Medical Photonics Research, Preeminent Medical Photonics Education and Research Center, Hamamatsu University School of Medicine, Hamamatsu, Japan; ^3^Department of Virology and Parasitology, Hamamatsu University School of Medicine, Hamamatsu, Japan

**Keywords:** two-photon microscopy, aberration correction, spatial light modulator, resolution enhancement, phase modulation, vortex beam, azimuthally polarization, multi-ring mask

## Abstract

In two-photon microscopy, aberration correction is an essential technique for realizing high resolution in deep regions. A spatial light modulator (SLM) incorporated into an optical system for two-photon microscopy performs pre-compensation on the wavefront of the excitation beam, restoring the resolution close to the diffraction limit even in the deep region of a biological sample. If a spatial resolution smaller than the diffraction limit can be achieved along with aberration correction, the importance of two-photon microscopy for deep region observation will increase further. In this study, we realize higher resolution observations in the deep region by combining two resolution-enhancement methods and an aberration correction method. Therefore, a z-polarizer is added to the aberration-correction optical system, and the SLM modulates the amplitude and phase of the excitation beam; in other words, complex-amplitude modulation is performed. The lateral resolution is found to be approximately 20% higher than the diffraction limit obtained using a circularly polarized beam. Verification was conducted by simulation and experimentation using model samples and *ex vivo* biological samples. The proposed method has the potential to be effective for live imaging and photostimulation of the deep region of the sample, although it requires only minor changes to the conventional optical system that performs aberration correction.

## Introduction

A near-infrared excitation beam that exhibits negligible scattering and absorption by biological samples is used in two-photon excitation fluorescence microscopy; thus, it has the potential to enable deep region observation, but the deterioration of image quality due to aberration during deep region observations cannot be ignored. For example, at the interface between the immersion fluid and the biological sample, spherical aberration (SA) occurs because of the change in the focusing angle of the excitation beam owing to the refractive index mismatch between the fluid and the sample (Gibson and Lanni, 1992; Torok et al., 1995; Booth et al., 1998; Escobar et al., 2006). The quasi-spherical focal shape of the excitation beam affected by SA extends on the optical axis, stretching the shape of the observed fluorescence in the optical axis because the intensity distribution of the fluorescence is proportional to the square of the intensity distribution of the focused excitation beam. Such deterioration of the two-photon point spread function (PSF) introduces a reduction in the resolution of the microscope in the deep region observation. Further, the elongation of the excitation beam introduces a reduction in the focusing density of the excitation beam which reduces the intensity of the emitted fluorescence, and hence, the signal-to-noise ratio. Because SA depends on the numerical aperture (NA) of the objective lens, even if a high NA objective lens is adopted for high resolution, the effect of SA intensifies further, significantly stretching the two-photon PSF in the axial direction. If the aberration correction is successful, the two-photon PSF can be restored to the diffraction limit, which is the original size. It has also been reported that the improvement in resolution through aberration correction can sharpen the observed image inside a biological sample (Ji et al., 2012). Thus far, phase modulation methods of the excitation beam for pre-compensation using various devices such as LCOS-SLM (Ji et al., 2010; Matsumoto et al., 2015; Yamaguchi et al., 2021), deformable mirrors (Turcotte et al., 2017), and objective lens correction collar (Ue et al., 2018) have been proposed. Among them, LCOS-SLM is inferior to deformable mirrors in terms of modulation speed but features the advantage of being able to represent complex wavefronts simply because of the large number of pixels. Thus, one SLM can simultaneously realize other functions in addition to aberration correction by utilizing this pixel-rich feature. For example, we have proposed a method to reduce the measurement time by realizing aberration correction and multi-focal generation simultaneously (Matsumoto et al., 2017), and another method to enhance the axial resolution by more than 8% above the diffraction limit in the deep region observation by simultaneously realizing aberration correction and amplitude-distribution-modulation (Matsumoto et al., 2021).

Although the two-photon PSF in the deep region is restored close to the diffraction limit by incorporating the SLM into the optical system, the two-photon PSF in the lateral direction is generally as low as 300–400 nm when an excitation beam with a wavelength of 890 nm is used in an upright microscope. This is due to the long wavelength of the excitation beam and the low NA of the commercially available objective lens with a long working distance. If the lateral resolution of the microscope is increased to approximately 200 nm, sub-cellular structures such as organelles and cytoskeletons can be observed in detail with resolution comparable to confocal microscopy (Konno et al., 2020). Thus, it is possible to detect the onset of cellular changes. However, because the NA of the objective lens has a physical limit and the excitation wavelength cannot be changed significantly, it is difficult to reach a lateral resolution of 250 nm or less.

In this study, we attempted to modulate the polarization, phase, and amplitude distributions of the excitation beam to achieve a lateral resolution of 250 nm or less, while retaining the possibility of deep penetration, which is a characteristic of two-photon excitation fluorescence microscopy. In particular, we modulate the spatial mode of the excitation beam by controlling the polarization distribution with a z-polarizer and by modulating the complex amplitude on the SLM. Here, using only one SLM, we employed two resolution-enhancement methods to obtain a resolution below the diffraction limit and an aberration correction method for deep region observation simultaneously. The first resolution-enhancement method uses an azimuthally polarized vortex excitation beam (Li et al., 2014). The focal shape of this excitation beam is a quasi-sphere, and it does not have an electric field in the axial direction (z-direction). The two-photon PSF in the lateral direction obtained by this excitation beam is shorter than that obtained by the circularly polarized excitation beam, particularly when an objective lens with a high NA is adopted. The polarization and phase of the excitation beam are modulated to generate an azimuthally polarized vortex beam. The second resolution-enhancement method further reduces the volume of the PSF by modulating the amplitude distribution of the azimuthally polarized vortex beam. An amplitude-modulation-type multi-ring mask for a circularly polarized beam (Matsumoto et al., 2021) is employed for amplitude modulation on the azimuthally polarized vortex beam. Because the focal shape changes three-dimensionally by the interference of the excitation beam that is divided in the radial direction, this method can enhance not only the lateral PSF but also the axial PSF. Phase modulation for aberration correction that is performed in conjunction with these resolution-enhancement methods, prevents the resolution degradation caused by SA, maintaining the enhanced resolution in the deep region of the sample. The combination of multiple methods may seem complicated, but by adopting the SLM, the only change in the optical system for aberration correction is the addition of the z-polarizer. This is because amplitude and phase distribution modulations using the SLM, that is, complex-amplitude modulation, does not involve any moving parts and simply adjusts alignment and changes the modulation pattern with simple electrical signal control. In other words, this method was employed to obtain the amplitude-distribution-modulated azimuthally polarized vortex excitation beam with aberration correction, and we performed observations with a resolution smaller than the diffraction limit obtained using the conventional circularly polarized beam. The experiment was performed using model samples and *ex vivo* biological samples. To confirm the effectiveness of combining the two resolution-enhancement methods, we compared the observed images obtained using the conventional circularly polarized excitation beam, excitation beams generated by each resolution-enhancement method, and excitation beam generated by a combination of the two resolution-enhancement methods in the shallow region of the sample. In the deep region of the sample, we confirmed the effectiveness of employing resolution-enhancement and aberration correction methods simultaneously. By combining the two resolution-enhancement methods, we show that the resolution-enhancement ratio is higher than that obtained by employing only one of the methods in every range examined for the NA of the objective lens. Further, we show that in this method, the higher the NA of the objective lens, the higher is the enhancement ratio in the lateral resolution from the diffraction limit.

In two-photon excitation fluorescence microscopy, the adoption of a near-infrared pulsed excitation beam with small scattering and aberrations caused by biological samples and an SLM that corrects aberration enables high-resolution observations in deep regions. This approach is advantageous over other super-resolution methods that use visible light. Furthermore, the active use of the SLM can enable the simultaneous implementation of the two resolution-enhancement methods that directly modify the focusing shape of the excitation beam and afford observation at a resolution below the diffraction limit in the deep region. Further, multiple measurements or complicated calculations are not required, and therefore, it is considered effective for *in vivo* measurements in the future.

## Materials and Methods

### Optical System

**FIGURE 1:**
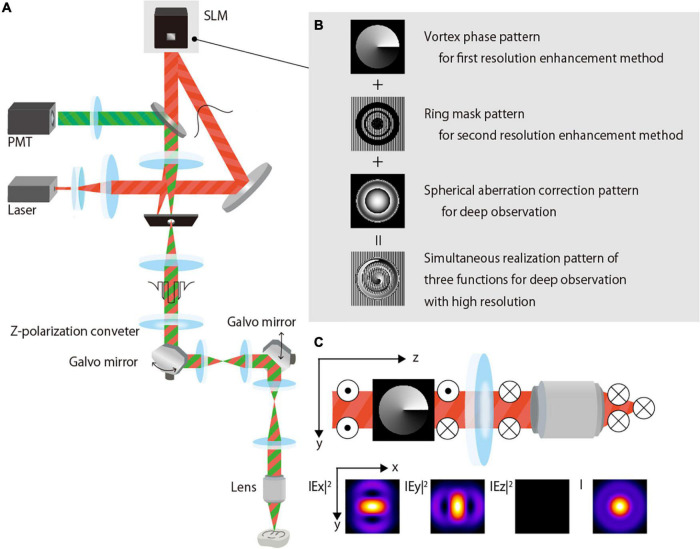
Proposed two-photon excitation fluorescence microscopy. **(A)** Schematic of the optical system setup. **(B)** Phase-modulation pattern for application in the SLM for the complex-amplitude modulation of the excitation beam. **(C)** Polarization and electric field of the azimuthally polarized vortex beam.

Figure 1A is a simplified schematic that shows the setup of our experimental two-photon excitation fluorescence microscope system for deep observation with a high resolution. An excitation beam (890-nm wavelength, 150-fs pulse duration, 80-MHz repetition rate) emitted from a Ti:sapphire laser (Chameleon Vision II, Coherent Inc., United States) is expanded by a beam expander and projected onto a phase-modulation-type LCOS-SLM (1272 × 1024 pixels, 12.5-μm pixel pitch, Hamamatsu Photonics K.K., Japan) with a Peltier system (Matsumoto et al., 2014). The SLM performs the complex amplitude modulation of the excitation beam and reflects the beam. The modulated excitation beam passes through the three telecentric relay lens systems and an x-y galvo scanner (6220Hm Cambridge Technology, NovantaPhotonics, United States); it reaches a 60× NA1.3 silicone immersion objective lens (UPlanSApo, Olympus Corp., Japan) attached to an upright microscope (BX63, Olympus Corp., Japan). The objective lens has a 300-μm working distance. The first telecentric relay lens system features an aperture with a large hole diameter that removes any undesired first-order beam generated when the amplitude distribution of the azimuthally polarized vortex beam is modulated by the SLM. A z-polarizer (RPC-900-06, Altechna, Lithuania) is also placed on the first telecentric relay lens system to convert the polarization of the excitation beam from horizontal to azimuthal. The excitation beam irradiates a sample using an objective lens, and the fluorescence is emitted from the irradiation position. The emitted fluorescence is collected by the objective lens and subsequently detected using a photomultiplier tube (H10770P-40, Hamamatsu Photonics K.K., Japan). A two-dimensional image of a plane perpendicular to the optical axis is obtained using a galvo scanner, and images of different depths are obtained by moving the objective lens in the optical axis direction. The obtained images are reconstructed into a three-dimensional image on a PC, and an x-y image or x-z projected image is developed, as required.

### Complex-Amplitude Modulation by the Spatial Light Modulator

To perform high-resolution observation in the deep region, the SLM modulates the excitation beam by applying a phase modulation pattern wherein three-phase modulation patterns are added (Figure 1B):

(i) Vortex phase pattern that is used to obtain the first resolution-enhancement method in combination with azimuthal polarization.
(ii) Amplitude-distribution-modulation-type ring-mask pattern that is used to perform the second resolution-enhancement method by modulating the amplitude distribution of the excitation beam, such that interference is actively used when the excitation beam is focused. A phase-modulation pattern with partially arranged gratings is used to represent the mask in the phase-modulation-type SLM (Figure 1B). The undesired first-order diffracted beam is cut off by a large-diameter aperture placed in the first telecentric relay lens system, and a zero-order beam with the desired amplitude distribution reaches the objective lens.
(iii) Aberration correction pattern that is used to obtain deep observations by correcting the spherical aberration caused by the refractive index mismatch between the immersion fluid and the sample.

The details of each pattern are provided in the following subsections. In addition, to correct aberrations other than SA, we added a pattern for correcting aberrations caused by the SLM obtained by a Michelson interferometer (Matsumoto et al., 2008); we also added a pattern for correcting aberrations inherent in the optical system obtained by image-based measurement (Bruke et al., 2015) in advance because the vortex phase is significantly affected by aberration. The correction patterns for the SLM and optical system were not changed during the experiment.

### First Resolution-Enhancement Method Based on Azimuthal Polarization and Vortex Phase

When both the beam with azimuthal polarization and the beam with vortex phase distribution are focused, the focused shape becomes a ring shape with a hole in the center (Supplementary Figure 1). The focused shape of the azimuthally polarized vortex beam generated by the two conversion elements, z-polarizer, and the SLM working as the vortex phase plate, is quasi-spherical without the hole because the polarization of the beam changes depending on each element (Figure 1C). Similarly, in the experiment using 0.2-μm-diameter fluorescence beads in a transparent epoxy resin, the shape of the observed bead is ring-shaped when each azimuthally polarized and vortex phase beam is used as the excitation beam (Supplementary Figure 2). However, when an azimuthally polarized vortex excitation beam is used, the shape of the observed bead is spherical. With the silicone immersion objective lens, the two-photon PSF of the azimuthally polarized vortex beam is smaller than that of the circularly polarized beam when the NA of the objective lens is 0.95 or more, and the resolution-enhancement ratio is higher when the NA is higher (Supplementary Figure 3).

### Second Resolution-Enhancement Method Based on Amplitude-Distribution-Modulation With Multi-Ring Mask

Modulating the amplitude distribution of the conventional circularly polarized excitation beam using a ring mask reduces the size of the two-photon PSF further (Matsumoto et al., 2021). Note that in this method, in addition to the desired fluorescence, undesired higher-order fluorescence is generated in the optical axis direction, and its intensity depends on the shape of the ring mask. Here, we call the desired fluorescence the main lobe and the undesired higher-order fluorescence the sidelobe. If the fluorescence intensity of the sidelobe is high, the quality of the obtained image deteriorates because the false image from the other depth is highly contaminated in the desired image. The simplest configuration is a single-ring mask that has one light-shielding region and two light-transmission regions. We have confirmed that the excitation beam modulated by this single-ring mask is significantly affected by SA, resulting in aberration-induced sidelobes and degradation of the quality of the obtained image. Therefore, to reduce the effect of SA, we developed a multi-ring mask with multiple light-shielding and light-transmission regions. In addition, the excitation beam modulated by the ring mask with a high-resolution enhancement ratio is generally affected by SA, but we discovered a multi-ring mask pattern that is less affected by SA while rendering a high-resolution enhancement ratio. Such a ring-mask pattern with a small effect of SA is useful in cases where the effect of SA correction is partial, resulting in residual SA.

In this study, a multi-ring mask applied to an azimuthally polarized vortex excitation beam was designed using vectorial diffraction calculations (Youngworth and Brown, 2000; Ohtake et al., 2008). Design parameters used in the calculation are as follows:

(i) The sidelobe intensity in the case of no aberration is set below 2.5% of the main lobe intensity.
(ii) The maximum allowable amount of residual SA is assumed to be the aberration occurring when the observation is performed at a depth of 25 μm in a biological sample with an average refractive index of 1.38. Even if SA occurs within this range, the aberration-induced side lobes do not have a significant effect on the acquired image.
(iii) The aberration-induced sidelobe intensity is set below 10% of the main lobe intensity.

A detailed design algorithm is presented in the Supplementary Material.

### Spherical Aberration Correction

Spherical aberration occurs when a medium with refractive index *n*_*2*_ different from the immersion fluid with refractive index *n*_*1*_ exists in the light path focused by an immersion objective lens. The wavefront aberration when an objective lens with NA moves inside the sample (at distance d from the interface) is expressed as


(1) $$\phi \,\left(\rho \right)=\frac{2\,\pi \,d}{\lambda }\,\left(\left(1+\eta \right)\,\sqrt{n_{2}^{2}-{\left(\bar{\mathrm{NA}}\,\rho \right)}^{2}}-\sqrt{n_{1}^{2}-{\left(\bar{\mathrm{NA}}\,\rho \right)}^{2}}\right),$$

where λ is the wavelength of the excitation beam, ρ is the normalized pupil radius, and η is the factor for changing the depth of the focal spot. The SA increases as the observation position deepens and that reduces the resolution, particularly in the axial direction. If pre-compensation is performed using the inverse phase distribution of Eq. 1, the focal shape without the effect of the SA is obtained at the desired position.

### Image Acquisition Method for Comparison

In the experiment, to compare the two-photon PSF of the circularly polarized beam and the azimuthally polarized vortex beam, the quarter-wave plate and z-polarizer were manually replaced. Furthermore, the phase distribution of the excitation beam was electrically modulated by the SLM to evaluate the two-photon PSF obtained *via* the amplitude-distribution-modulation with a multi-ring mask and aberration correction.

### Animal Samples

Thy1-YFP-h mice with a significant presence of expressing yellow fluorescent protein (YFP) in the motor neurons (Feng et al., 2000) were obtained from the Jackson Laboratory (United States). They were kept under specific-pathogen-free conditions on a 12-h dark/light cycle with food and water *ad libitum* until use. All animal experiments were approved by the Institutional Animal Care and Use Committee of Hamamatsu University School of Medicine (Permission no.: 2019009) and were performed following the relevant guidelines and regulations. A one year and 6 months-old female heterozygous Thy1-YFP-H mouse was anesthetized using a mixed anesthetic (Kawai et al., 2011) and subsequently perfusion-fixed with 4% PFA in 0.1-M phosphate buffer (pH 7.4). The brain was isolated and fixed in the same fixative for 1 h at room temperature (approximately 25°C). After several washes with PBS, images were obtained *via* two-photon microscopy.

## Simulation Results

In this study, two resolution-enhancement methods were adopted and used in combination. To demonstrate the advantages of combining the two resolution-enhancement methods, four conditions were simulated: conventional circularly polarized (CP) excitation beam not employing the resolution-enhancement methods, azimuthally polarized vortex (APV) excitation beam with the first resolution-enhancement method, amplitude-distribution-modulated circularly polarized (AMCP) excitation beam with the second resolution-enhancement method, and amplitude-distribution-modulated azimuthally polarized vortex (AMAPV) excitation beam generated by employing both the first and second resolution-enhancement methods. We compared them and found that the AMAPV excitation beam was the most effective.

**FIGURE 2:**
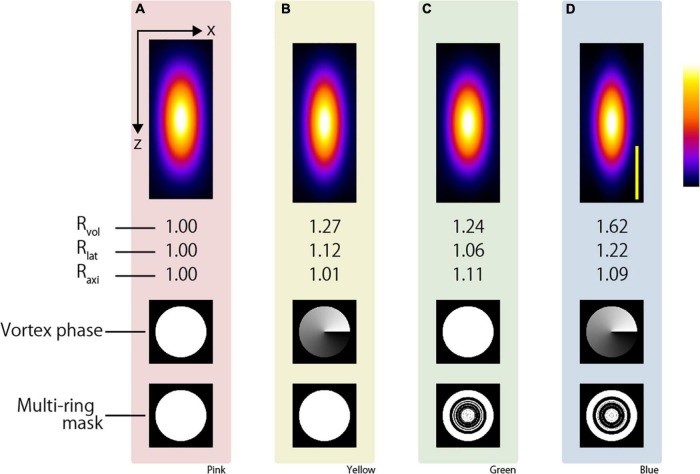
Simulation result of two-photon PSF when the silicone immersion objective lens with NA = 1.3 was used to focus the **(A)** CP, **(B)** APV, **(C)** AMCP, and **(D)** AMAPV excitation beams. Top: an x-z projected images of the two-photon PSF. Middle: phase patterns for realizing the first resolution-enhancement method. White and black indicate a phase difference of 0 to 2π. Bottom: multi-ring mask patterns for realizing the second resolution-enhancement method. White and black areas represent the transmission and light-shielding areas, respectively. Scale bar: 500 nm. Similarly in the following figure, the images surrounded by pink, yellow, green, and blue backgrounds show the results obtained using the CP, APV, AMCP, and AMAPV excitation beams, respectively.

First, we compared the two-photon PSFs of the CP and APV excitation beams *via* simulation. Here, we evaluated the two-photon PSF, i.e., the size of fluorescence emitted from a fine fluorescent object when the excitation beam with a top-hat intensity distribution and an 890-nm wavelength irradiated the object through an NA 1.3 silicone objective lens. Under the present condition, the shape of the two-photon PSF was an ellipsoidal sphere whose axial direction (optical axis) was more than twice as long as the lateral direction (perpendicular to the optical axis). To evaluate the size of the two-photon PSF, the volume V of the two-photon PSF was calculated from the area with a fluorescent intensity greater than half of the maximum fluorescent intensity as


(2) $$V={\left(L_{l\,a\,t}/2\right)}^{2}*\left(L_{a\,x\,i}/2\right),$$

where *L*_*lat*_ and *L*_*axi*_ denote the full width at half maximum in the lateral and axial directions, respectively. In addition, the volume enhancement ratio *R*_*vol*_ is calculated as the volume obtained with the CP excitation beam divided by the volume obtained with the excitation beam applying the resolution-enhancement method. The lateral resolution-enhancement ratio *R*_*lat*_ and axial resolution-enhancement ratio *R*_*axi*_ are calculated in the same manner. If the two-photon PSF obtained by the resolution-enhancement method becomes small, these values will be greater than 1. Figures 2A,B show the x-z-projected two-photon PSF images obtained using the CP excitation beam and the APV excitation beam, respectively. When the APV excitation beam was used, it was calculated as *R*_*lat*_ = 1.12 and *R*_*axi*_ = 1.01, and consequently, *R*_*vol*_ = 1.27 was obtained.

Next, for both the AMCP and AMAPV excitation beams, we individually designed eight-ring masks that minimized the volume of the two-photon PSF. Note that the optimal ring mask shape is different for the AMCP and AMAPV excitation beams although they are designed using the same algorithm (Figures 2C,D and Supplementary Material); this is because the AMCP tends to specifically enhance the PSF in the axial direction, whereas the AMAPV tends to specifically enhance the PSF in the lateral direction; thus, it automatically generates different patterns. Figures 2C,D show the x-z-projected two-photon PSF images obtained using the AMCP and AMAPV excitation beams, respectively. *R*_*lat*_ and *R*_*axi*_ were 1.06 and 1.11, respectively, when using the AMCP excitation beam; consequently, *R*_*vol*_ was calculated as 1.24. The volume enhancement ratio obtained by the AMCP excitation beam was equivalent to that obtained by the APV excitation beam. However, the axial resolution-enhancement ratio was higher than the lateral resolution-enhancement when the AMCP excitation beam was used, and the opposite trend was observed for the APV excitation beam. The volume and lateral PSF were the smallest among the four conditions when the AMAPV excitation beam was used. In this case, *R*_*lat*_ and *R*_*axi*_ were calculated as 1.22 and 1.09, respectively, and consequently, *R*_*vol*_ was determined to be 1.62. Radial polarization is another well-known vectorial polarization, but the volume of the PSF was the smallest when the AMAPV excitation beam was used (Supplementary Figure 4). Under the conditions of using an 890-nm wavelength excitation beam and a silicone immersion objective lens with NA = 1.3, the lateral and axial PSFs were 276 and 669 nm, respectively, when the CP excitation beam was used, and the lateral and axial PSFs were enhanced to 226 and 614 nm, respectively, when the AMAPV excitation beam was used.

## Experimental Results

### Model Samples

First, we performed measurements using model samples wherein the size of the fluorescent material enclosed in the sample was known in advance. We investigated the effects of combining the resolution-enhancement methods in the shallow region observation and of combining them with aberration correction in the deep region observation, respectively.

#### Observation in the Shallow Region of Model Sample

**FIGURE 3:**
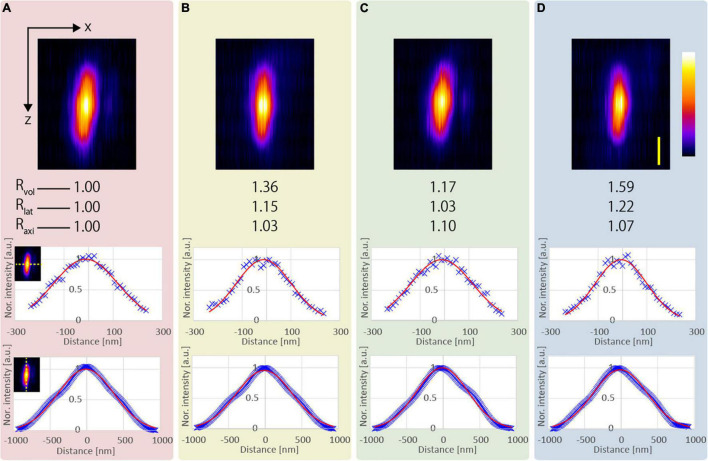
Observation results of a fluorescent nanodiamond particle existing near the surface of the epoxy resin obtained using the **(A)** CP, **(B)** APV, **(C)** AMCP, and **(D)** AMAPV excitation beams. Top: the x-z projected images of the nanodiamond. Middle: line profiles and Gaussian fittings in the lateral direction. Bottom: line profiles and Gaussian fitting results in the axial direction. The excitation beam intensity under the objective lens was **(A)** 18.7, **(B)** 22, **(C)** 25.7, and **(D)** 28.2 mW. Scale bar: 500 nm.

We observed five functionalized fluorescent nanodiamond particles (NDNVNsyn120 nm, Adámas Nanotechnologies, United States) with an average particle size of 110 nm placed in a transparent epoxy resin (average refractive index *n* = 1.55). Figure 3 shows an example of the x-z projected images of the observed nanodiamond near the surface of the epoxy resin. All observations applying the resolution-enhancement methods demonstrated a higher resolution than those obtained using the conventional CP excitation beam. Further, Figure 3 shows that the lateral FWHM of the observed nanodiamond using the APV excitation beam was 15% smaller than that of the observed nanodiamond using the CP excitation beam. Furthermore, in the observation using the AMCP, the axial FWHM of the observed nanodiamond was the smallest, and Δ*L*_*axi*_ = 1.10 was obtained (Figure 3C). In Figure 3D, by using the AMAPV excitation beam, the lateral FWHM of the observed nanodiamond was the smallest, and *R*_*lat*_ = 1.22 was obtained. The axial FWHM of the observed nanodiamond using the AMAPV excitation beam was worse than that using the AMCP, but Δ*L*_*axi*_ was still obtained as 1.07.

Table 1 shows the evaluation results obtained after the measurement of the five nanodiamond particles. Compared with the result obtained using the CP excitation, *R*_*lat*_ is enhanced by an average of 21% when the AMAPV excitation beam is used, and this experimental result is equivalent to the simulation result of 23%. Similarly, Δ*L*_*axi*_ is enhanced by an average of 8% when the AMAPV excitation beam is used. For the evaluation of the subsequent experiments, Table 2 shows the comparison results of the APV and AMAPV excitation beams. Because of amplitude-distribution-modulation, the horizontal and axial lengths of the observed nanodiamond obtained using the AMAPV excitation beam were 12 and 3%, respectively.

**TABLE 1:** Resolution-enhancement ratio compared with the CP excitation beam after the measurement of five nanodiamond particles.

	Experimental result (Standard deviation)		Simulation result	
	*R*_lat_	*R*_axi_	*R*_lat_	*R*_axi_
APV excitation beam	1.10 (0.03)	1.04 (0.02)	1.12	1.02
AMCP excitation beam	1.06 (0.03)	1.12 (0.02)	1.06	1.11
AMAPV excitation beam	1.21 (0.02)	1.08 (0.01)	1.23	1.06

**TABLE 2:** Effect of applying the eight-ring mask to modulate the amplitude distribution of the APV excitation beam.

	Experimental result (Standard deviation)		Simulation result	
	Lateral direction	Axial direction	Lateral direction	Axial direction
Change from APV to AMAPV	1.12 (0.03)	1.03 (0.02)	1.09	1.06

*The values indicate the resolution-enhancement ratio when changing from the observation using the APV excitation beam to the observation using the AMAPV excitation beam.*

*The comparison was made using the measurement of five nanodiamond particles.*

#### Observation in the Deep Region of Model Samples

**FIGURE 4:**
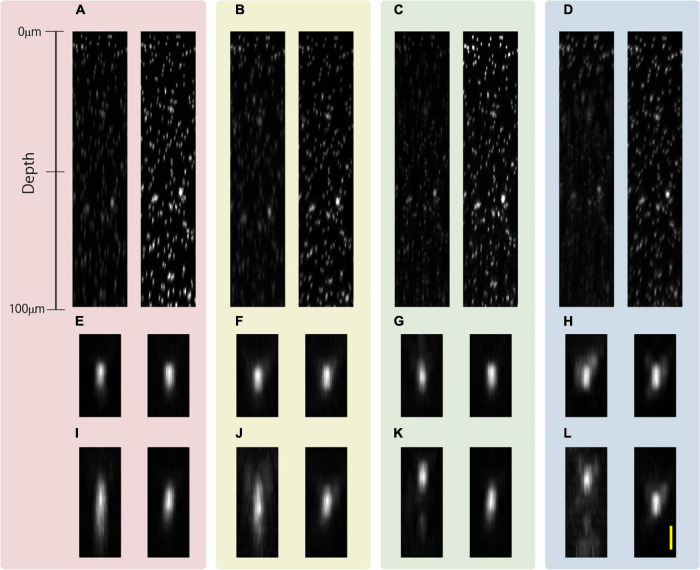
x-z projected images of 500-nm-diameter fluorescent beads in a transparent epoxy resin obtained using the **(A)** CP, **(B)** APV, **(C)** AMCP, and **(D)** AMAPV excitation beams. Left: without SA correction. Right: with SA correction. **(E–H)** Magnified view of the observed bead existing near the surface of the epoxy resin. **(I–L)** Magnified view of the observed bead existing at an optical depth of approximately 80 μm. Scale bar: 1000 nm.

Aberration correction is necessary because SA occurring during deep observation significantly deteriorates resolution, even if resolution-enhancement methods are employed. The observation conducted based on each resolution-enhancement method can reduce the effect of the SA owing to the application of an aberration correction pattern using the wavefront calculated by Eq. 1. To show that the image quality is improved simply by aberration correction when using the excitation beam generated by the resolution-enhancement method as when using the CP excitation beam, we observed 500-nm-diameter fluorescent beads in transparent epoxy resin with and without aberration correction. The aberration correction pattern was redesigned with every 1.0-μm movement of the objective lens. The intensity of the excitation beam was changed according to the depth, such that the fluorescence intensity was larger than the noise even when observed in the deep region. The intensity of the excitation beam was different for the observation based on each resolution-enhancement method, but the same intensity was used for comparison between the observations based on the method with and without SA correction. Figure 4 shows the x-z projected images from an optical depth of 0–100 μm. The left side of Figure 4A shows the image obtained using the CP excitation beam without SA correction, and the right side of Figure 4A shows the image obtained using the CP excitation beam with SA correction. Similarly, Figures 4B–D show images obtained using the APV, AMCP, and AMAPV excitation beams, respectively. In addition, Figures 4E–H show magnified views of the bead existing near the surface, and Figures 4I–L show the magnified views of the bead obtained at an optical depth of 80 μm. First, we explain the observation results without SA correction. Figures 4A,B show that the elongation in the axial direction of the observed bead increased as the observation depth increased in both observations obtained using the CP and APV excitation beams. When the resolution-enhancement methods using the ring mask are performed, a ghost image of a bead is observed in the axial direction in the deep region when resolution-enhancement methods using the ring mask are employed (Figures 4C,D). The reason is that the residual SA causes the excitation beam to be double focused if the SA exceeds the allowable amount of aberration in the resolution-enhancement method using the ring mask. Next, we explain the observed results with SA correction. As expected, deep observation was possible by simply applying the SA pattern according to Eq. 1 in any excitation beam condition. The elongation in the axial direction of the observed image of the bead in the deep region was suppressed, and the shape of the bead was similar to that of the bead observed near the surface. In addition, because the energy density of the focused excitation beam is also improved by SA correction, the fluorescent intensity of the observed bead with SA correction was higher than that of the observed bead without SA correction in the deep region. Thus, we confirmed that SA correction can be applied to the resolution enhancement-methods in the deep region.

**FIGURE 5:**
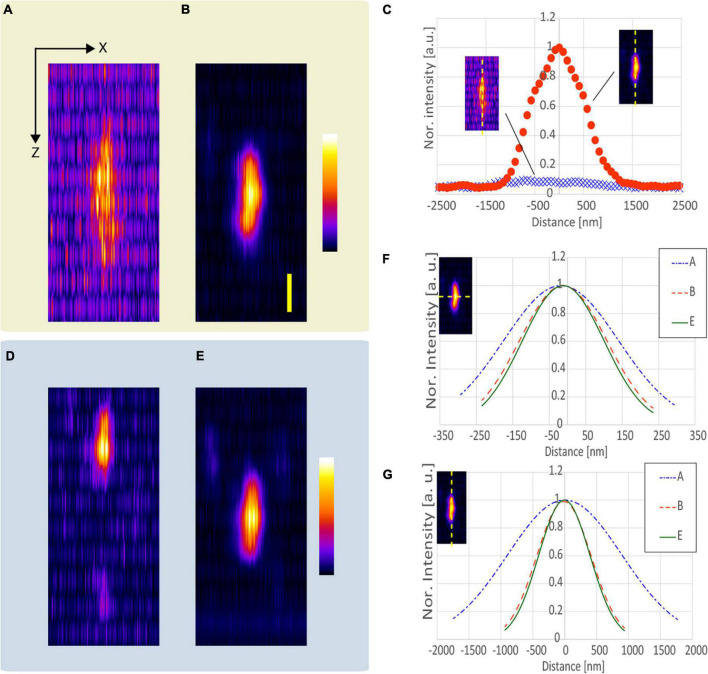
Observation results of a fluorescent nanodiamond existing at an optical depth of 53.7–57.1 μm. The x-z projected images of the nanodiamond obtained using the APV, **(A)** without and **(B)** with SA correction. **(C)** Axial profile of the nanodiamond obtained using the APV excitation beam with and without SA correction. The x-z projected images obtained using the AMAPV excitation beam **(D)** without and **(E)** with SA correction. **(F)** Gaussian fitting results in the lateral direction. **(G)** Gaussian fitting results in the axial lateral direction. The excitation beam intensity under the objective lens was **(A,B)** 38.7 and **(D,E)** 69 mW. Scale bar: 500 nm.

Subsequently, we evaluated the lengths of the observed fluorescent nanodiamond in the epoxy resin obtained with and without SA correction when APV and AMAPV excitation beams were used. We focused on the intensity of the excitation beam such that the size of the observed bead would not be overly magnified by fluorescent saturation. Figures 5A,B show the x-z projected images of the observed nanodiamond when observations using the APV excitation beam with and without SA correction were performed from an optical depth of 53.7–57.1 μm. From the axial profile shown in Figure 5C, SA correction improved fluorescence intensity by a factor of 10.5. Similarly, observations using the AMAPV excitation beam with and without SA correction were performed (Figures 5D,E). Figures 5F,G show Gaussian fitting results in the lateral and axial directions of the obtained nanodiamond (details of the measured values and fitting are shown in Supplementary Figure 5). When using the APV excitation beam, the lateral and axial lengths of the observed nanodiamond obtained with SA correction were 1.32 and 2.11 times shorter than those obtained without SA correction, respectively. After applying SA correction, the lateral and axial lengths of the observed nanodiamond obtained using the AMAPV excitation beam were 1.07 and 1.06 times shorter than those obtained using the APV excitation beam, respectively. This result was almost the same as the simulation results summarized in Table 2.

### *Ex vivo* Samples

**FIGURE 6:**
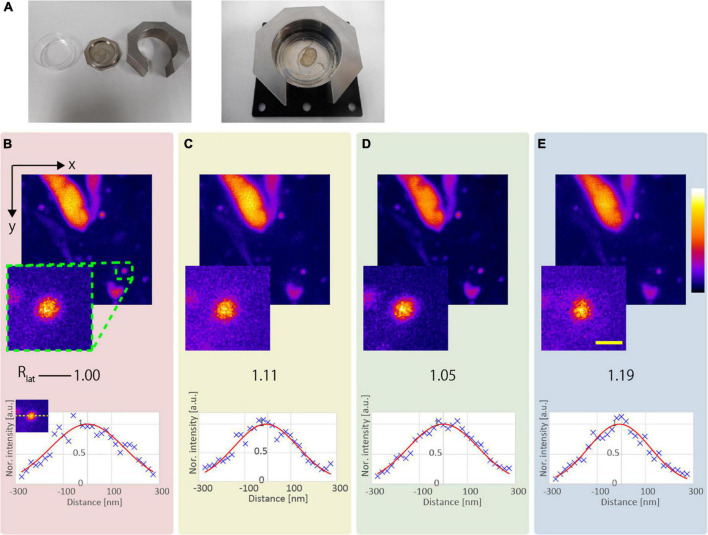
Observation result of the YFP mouse brain near the surface. **(A)** Photograph of the developed fixing tool. **(B)** CP, **(C)** AMCP, **(D)** APV, and **(E)** AMAPV excitation beams. Top: x-y stacked images from 0 to 9 μm optical depth. Bottom: line profile and Gaussian fitting result in the lateral direction. The excitation beam intensity under the objective lens was **(B)** 17.1, **(C)** 28.8, **(D)** 23.8, and **(E)** 30.3 mW. Scale bar: 500 nm.

We evaluated the resolution-enhancement effect of the proposed method using a fixed mouse brain as the biological sample. High-NA objective lenses generally have coverslip correction for observation using an inverted microscope. Because the proposed method is more effective when the NA of the objective lens is larger, we developed a fixing tool (Figure 6A), and observed the sliced mouse brain through the coverslip.

#### Observation Near the Surface of *ex vivo* Sample

Figures 6B–E show the x-y stacked images obtained from an optical depth of 0–9 μm. All observations obtained by applying the resolution-enhancement methods had a higher resolution than the observation using the conventional CP excitation beam. When AMCP is used, *R*_*lat*_ = 1.05 (Figure 6D). By using the APV excitation beam, *R*_*lat*_ = 1.11 is obtained, and the observed structure became smaller (Figure 6C). Furthermore, when the AMAPV beam is used as the excitation beam, we obtain *R*_*lat*_ = 1.19, which is equivalent to the simulation value of 1.23 (Figure 6E).

**FIGURE 7:**
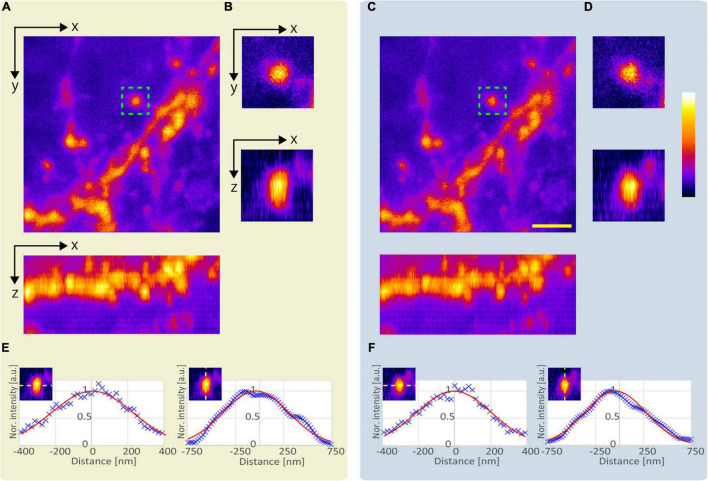
Observation result of the YFP mouse brain at an optical depth from 19.6 to 23.7 μm. **(A)** Images obtained using the APV excitation beam. Top: x-y stacked image. Bottom: x-z projected image. **(B)** Magnified views of the area surrounded by green dash squares in panel **(A)**. **(C)** Images obtained using the AMAPV excitation beam. **(D)** Magnified views of the area surrounded by green dash squares in panel **(C)**. **(E,F)** Line profile and Gaussian fitting result. The excitation beam intensity under the objective lens was **(A)** 53.3 mW and **(C)** 71.4 mW. Scale bar: 2 μm.

Next, we compared the lateral and axial lengths of the structures obtained using the APV and AMAPV excitation beams at an optical depth from 19.6 to 23.7 μm. Figure 7A shows the x-y and x-z stacked images obtained using the APV excitation beam. Figure 7B shows the magnified views of the area surrounded by the green dashed squares in Figure 7A. Figures 7C,D were obtained using the AMAPV excitation beam. Figures 7E,F show the line profile and Gaussian fitting result, and the lateral and axial lengths of the observed structure by applying the ring mask were shortened by 1.09 and 1.06 times, respectively. The resolution-enhancement ratio is the same as the simulation result summarized in Table 2.

#### Observation in the Deep Region of *ex vivo* Sample

**FIGURE 8:**
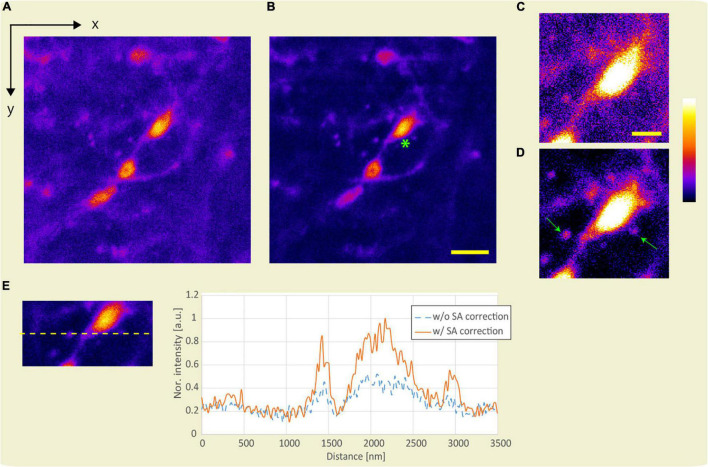
Observation result of the YFP mouse brain at an optical depth of 186.5 μm. x-y images were obtained using the APV excitation beam **(A)** without and **(B)** with SA correction. Scale bar: 5 μm. **(C,D)** Magnified view of the area around the green asterisk in panel **(B)**. Scale bar: 2 μm. **(E)** Line profile. The excitation beam intensity under the objective lens is 172 mW.

Applying SA correction for observation in the deep region of the sample improves the intensity and size of the emitted fluorescence. First, we demonstrate improvement in fluorescence intensity by SA correction. Figures 8A,B show the x-y images obtained using the APV excitation beam with and without SA correction at an optical depth of 186.5 μm. Assuming the average refractive index of the sample to be 1.38, the SA correction pattern was calculated using Eq. 1. Figures 8C,D show magnified views of the area around the green asterisk in Figures 8A,B. Note that the same image processing technique was applied to Figures 8C,D. Because the fluorescent intensity was improved by SA correction, dendric spines in Figure 8D are observed compared to those in Figure 8C (green arrows). Figure 8E shows the line profiles with and without SA correction. By applying SA correction, the fluorescence intensity was improved by 1.82 times.

**FIGURE 9:**
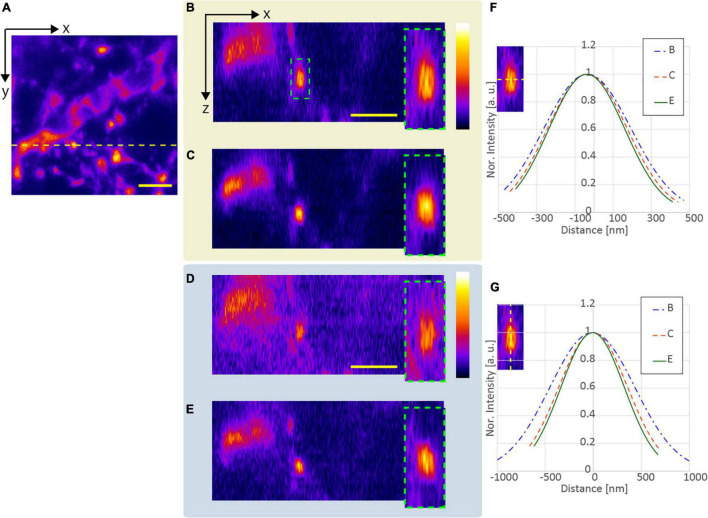
Observation result of the YFP mouse brain at an optical depth of 75.5–81.9 μm. **(A)** x-y stacked image obtained using the APV excitation beam. The x-z images on the yellow dotted line (panel **A**) when using the **(B)** APV excitation beam without SA correction, **(C)** APV excitation beam with SA correction, **(D)** AMAPV without SA correction, and **(E)** AMAPV with SA correction. **(F)** Gaussian fitting results in the lateral direction. **(G)** Gaussian fitting results in the axial lateral direction. The excitation beam intensity under the objective lens was **(B,C)** 144 and **(D,E)** 162 mW. Scale bar: 3 μm.

Subsequently, to confirm the effect of resolution-enhancement in the deep region, we evaluated the lengths of the observed fluorescent structures obtained with and without SA correction when the APV and AMAPV excitation beams were used. Figure 9A shows the x-y stack image at an optical depth from 75.5 to 81.9 μm. Figures 9B,C show the x-z images on the yellow dotted line shown in Figure 9A when the APV excitation beams with and without SA correction are used. Magnified views of the area surrounded by the green dashed squares are also shown. Figures 9D,E show the x-z images obtained using the AMAPV excitation beam with and without SA correction, respectively. Figures 9F,G show the Gaussian fitting results in the lateral and axial directions of the observed structure (The details of the measured values and the fitting are shown in Supplementary Figure 6). When using the APV excitation beam, the lateral and axial lengths of the observed structure obtained with SA correction were 1.08 and 1.28 times shorter than those obtained without SA correction, respectively. Moreover, by changing the excitation beam from APV to AMAPV, the lateral and axial lengths were further shortened by 1.05 and 1.05 times, respectively. This experimental enhancement ratio owing to the change in the excitation beam was almost the same as the simulation result summarized in Table 2.

## Discussion

The evaluation of SA correction using the model sample was conducted at an optical depth of 50 μm. This is because the accuracy of the resolution-enhancement ratio may be degraded if the evaluation is conducted at a deeper depth. For observations at a depth deeper than 50 μm, we considered that the fluorescence intensity obtained without SA correction would be lower than the noise level if the intensity of the excitation beam was low, or the fluorescence intensity obtained with SA correction would saturate if the intensity of the excitation beam was high. If we do not consider the accuracy of the quantitative comparison, the proposed method can be applied for observations deeper than 50 μm.

The improvement ratio of the fluorescent intensity with SA correction was significantly different between the evaluations using the model sample and the biological sample because of the difference in the refractive indices of the samples. The refractive index difference between the biological sample and silicone oil (immersion fluid, average refractive index of 1.406) is considerably smaller than that between the silicone and epoxy resin used in the evaluation of the model samples. Although the effect of SA appeared to be minimal during the biological observation, the SA halved the fluorescent intensity and reduced the resolution in the axial direction by approximately 30% (Figures 8, 9).

The method for designing the SA correction pattern *via* calculation is based on the average refractive index of the biological sample. Because the calculation was not based on the sample shape or the exact distribution of the refractive index, precise aberration correction was difficult and residual aberrations remained. Therefore, we adopted a multi-ring mask that reduces the effect of residual aberration for amplitude-distribution-modulation. The aberration correction patterns that use calculations considering the exact distribution of the refractive index and adaptive optics with wavefront sensors (Wang et al., 2015; Turcotte et al., 2017) may further reduce the effects of aberrations. In this case, we apply the multi-ring mask that provides higher resolution, and consequently, we can expect an increase in the resolution in the axial direction. The use of SLM that has a large number of pixels and high diffraction efficiency extends the depth of observation with high resolution. However, the optical setup in Figure 1 may need to be slightly modified depending on the light utilization efficiency of the SLM. In this study, an SLM with high light-utilization efficiency was adopted. Due to this high light-utilization efficiency and the relationship that the intensity of fluorescence is proportional to the square of the intensity of the excitation beam, the difference between the fluorescence excited by controllable and uncontrollable zero-order beam became large. As a result, the effect of the uncontrollable zeroth-order light could be ignored; and the controllable zero-order beam was actively used. However, pixel size tends to decrease with an increase in the number of pixels; this reduces the ratio of pixel size to pixel gap, resulting in reduced SLM light-utilization efficiency. Thus, the effect of the unmodulated zero-order light becomes stronger and cannot be ignored. In such cases, the effect of the uncontrolled zero-order beam effect can be eliminated by using the first-order beam as excitation beam and passing the beam diffracted by the SLM through a large aperture.

In our experiments, it was required that the excitation intensity of the APV excitation beam was set to 1.4–1.7 times higher than that of the CP excitation beam to achieve the same level of fluorescent intensity. The simulation clarified that the peak intensity of the APV excitation beam was reduced to 68% of that of the CP excitation beam. On the other hand, the following was also assumed: The intensity of the emitted fluorescence is the strongest when the orientation of the fluorescent molecule coincides with the polarization direction of the excitation beam. Because the APV excitation beam does not have polarization in the z-direction, it was expected that the fluorescent intensity when the APV excitation beam is used will be lower than that when the CP excitation beam is used. However, the effect of the polarization of the excitation beam was not noticeable. We consider that the small effect of the difference in polarization on the fluorescence intensity is attributed to the fluctuation in the fluorescence molecules in the sample.

**FIGURE 10:**
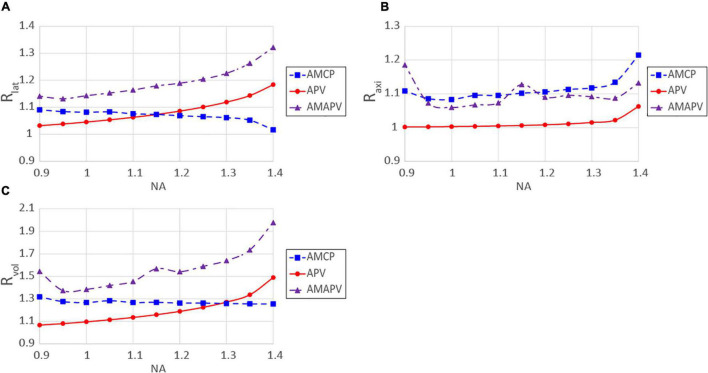
Resolution-enhancement ratios obtained for each NA; **(A)** *R*_*lat*_, **(B)** *R*_*axi*_, **(C)** *R*_*vol*_. The excitation beam was focused through the silicone immersion objective lens.

In this study, we used a combination of two resolution-enhancement methods that are particularly advantageous when performing observations using an objective lens with a high NA. Figure 10 shows the change in *R*_*lat*_, *R*_*axi*_, and *R*_*vol*_ for each NA when the excitation beam was modulated by two resolution-enhancement methods and focused on by the silicone immersion objective lens. When the AMCP excitation beam was used, *R*_*lat*_ and *R*_*vol*_deteriorated as NA increased. In the case of the APV excitation beam, *R*_*lat*_ increases as NA increases, but *R*_*axi*_ does not change significantly as NA changes. Moreover, *R*_*vol*_ obtained using the APV excitation beam was lower than that obtained using the AMCP excitation beam when an objective lens with a low NA was used. For these results, when the AMAPV excitation beam was used, both *R*_*lat*_ and *R*_*axi*_ increased above 15% or more, and *R*_*vol*_ was the highest in every range examined for the NA of the objective lens (NA ≥0.95). Based on the simulation, with a NA 1.35 silicone immersion objective lens (with the largest NA of the silicone immersion objective lens) adopted, *R*_*lat*_ and *R*_*axi*_ obtained using the AMAPV excitation beam were 1.27 and 1.14 times larger than those obtained using the CP excitation beam. Consequently, *R*_*vol*_ = 1.83 was obtained. Even if the beam with an 890 nm wavelength is used as the excitation beam, it is expected that the two-photon PSFs in the lateral and axial directions will be 211 and 514 nm, respectively.

**FIGURE 11:**
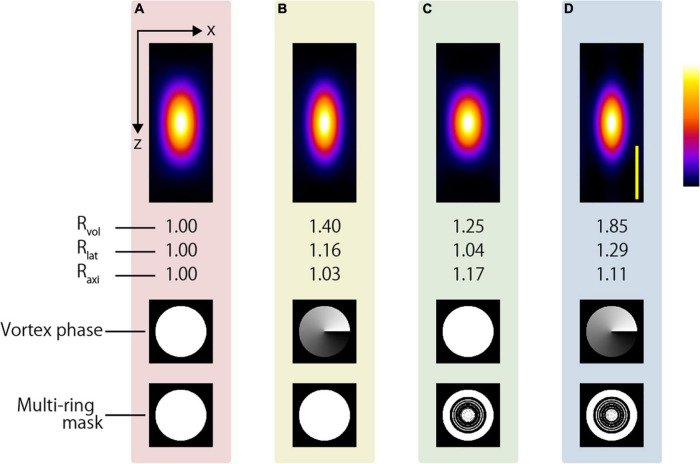
Simulation results of two-photon PSFs when the oil immersion objective lens with NA = 1.49 is used to focus using the **(A)** CP, **(B)** APV, **(C)** AMCP, and **(D)** AMAPV excitation beams. Top: the x-z projected images of the two-photon PSFs. Middle row: phase patterns for realizing the first resolution-enhancement method. Bottom: eight-ring mask pattern for realizing the second resolution-enhancement method. Scale bar: 500 nm.

This tendency was the same even when an oil-immersion objective lens was used. Although there is a limitation in that only the surface is observed, an even higher resolution-enhancement ratio was expected when the AMAPV excitation beam was focused using an oil immersion objective lens whose NA was 1.49. Figure 11 shows the x-z projected two-photon PSF images obtained using the oil-immersion objective lens with an NA of 1.49. The two-photon PSFs in the lateral and axial directions were 246 and 491 nm when the CP excitation beam was used, while those were 190 and 444 nm, respectively, when the AMAPV excitation beam was used.

**FIGURE 12:**
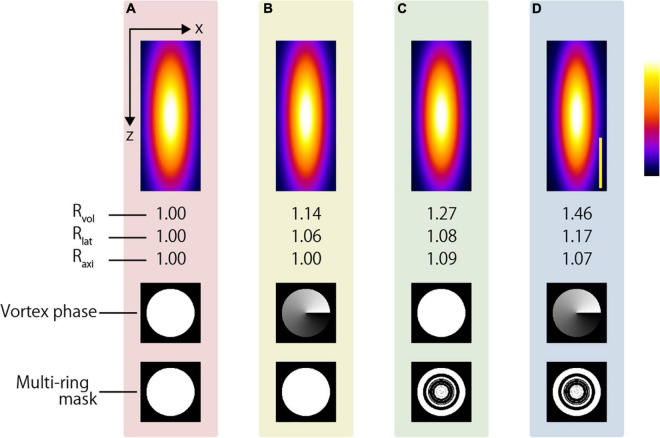
Simulation results of two-photon PSFs when the water immersion objective lens with NA = 1.05 is used to focus using the **(A)** CP, **(B)** APV, **(C)** AMCP, and **(D)** AMAPV excitation beams. Top: the x-z projected images of the two-photon PSFs. Middle row: phase patterns for realizing the first resolution-enhancement method. Bottom: eight-ring mask pattern for realizing the second resolution-enhancement method. Scale bar: 500 nm.

In this study, we showed the possibility of observing the deep region with a resolution below 250 nm *via* simple modifications to the optical system for aberration correction. A water immersion objective with a slightly smaller NA than that used in this study is utilized when performing live imaging for a long working distance. Figure 12 shows the simulation results using a 1.05 NA water immersion objective lens (XLPLN25XWMP2, Olympus). The objective lens has a 2000-μm working distance, and *R*_*lat*_ and *R*_*axi*_ obtained using the AMAPV excitation beam are 1.17 and 1.07 times larger than those obtained using the CP excitation beam. The two-photon PSFs in the lateral and axial directions were 330 and 1116 nm when the CP excitation beam was used, and 283 and 1038 nm, respectively, when the AMAPV excitation beam was used. The proposed method is expected to enhance the lateral resolution by approximately 50 nm; however, to obtain this resolution in the deep region, it is necessary to correct aberration with high accuracy even if large aberration occurs.

## Conclusion

We aimed at enhancing the resolution, which is a challenge in two-photon microscopy. The results obtained in our study revealed the following:

1) By adopting two resolution-enhancement methods that can be used in two-photon microscopy and do not affect each other, high-resolution observation combined with aberration correction is achievable in the deep region.
2) The proposed method, achieved by modulating the polarization, phase, and amplitude distributions of the excitation beam afforded a higher enhancement in the resolution, and it was applicable to observations using a low to high NA objective lens compared with the conventional CP excitation beam, and the beam generated based on every single method.
3) The proposed method enhances the lateral and axial PSFs. It has the potential to obtain a lateral PSF of approximately 226 nm.

4) Although the biological sample is observable using the proposed method, coupling it with aberration correction in deep region observation is necessary.
5) By adopting the SLM, the only change in the optical system for aberration correction is the addition of the z-polarizer. The SLM conducts both aberration correction and resolution-enhancement methods simultaneously, which are advantageous for deep observation with high resolutions.

The two-photon excitation observation using the upright microscope enabled deep region observation, but because the NA of the commercial water immersion objective lens was as low as 1.1, and therefore, the resolution in the lateral and axial directions using the CP excitation beam with 890-nm wavelength were 317 and 988 nm, respectively. Thus, we developed a fixed tool to use an objective lens with high NA for inverted microscopy, and we performed the observation with high resolution. The lateral and axial resolutions of the conducted two-photon microscopy were 226 and 614 nm, respectively, using the silicone immersion objective lens with NA = 1.3 and the AMAPV excitation beam. The proposed method directly modifies the two-photon PSF, and the SLM can simultaneously generate multiple focal points with a small two-photon PSF (Nikolenko et al., 2008; Matsumoto et al., 2017; Yang et al., 2018). The potential applications of the proposed method include multi-focal simultaneous observation and multi-point simultaneous photostimulation that can further contribute to the study of biological systems. To realize the resolution-enhancement method described above, even in the deep region of the sample, it is essential to remove the aberration sufficiently. For this reason, we believe that further development of aberration correction will significantly contribute to higher-resolution observations in the deep region.

## Data Availability Statement

The original contributions presented in the study are included in the article/Supplementary Material, further inquiries can be directed to the corresponding author.

## Ethics Statement

The animal study was reviewed and approved by the Institutional Animal Care and Use Committee of Hamamatsu University School of Medicine.

## Author Contributions

NM performed all measurements and drafted the manuscript. KW discussed the resolution-enhancement method using polarization and phase control techniques. TI organized the research and discussed the aberration-correction method. AK provided the biological samples and discussed the resolution required for the biological measurements. SO organized the research and provided the biological and model samples. All authors reviewed and approved the final manuscript.

## Conflict of Interest

The key device, SLM, was developed by Hamamatsu Photonics K.K. Although NM, TI, and KW are affiliated with Hamamatsu Photonics K.K., they have conducted the experiments in good faith. The remaining authors declare that the research was conducted in the absence of any commercial or financial relationships that could be construed as a potential conflict of interest.

## Publisher’s Note

All claims expressed in this article are solely those of the authors and do not necessarily represent those of their affiliated organizations, or those of the publisher, the editors and the reviewers. Any product that may be evaluated in this article, or claim that may be made by its manufacturer, is not guaranteed or endorsed by the publisher.
