# Supplementary material for: Complex-Amplitude-Modulation Vectorial Excitation Beam for High-Resolution Observation of Deep Regions in Two-Photon Microscopy
Source: Front Neurosci. 2022 Apr 19;16:880178. doi: 10.3389/fnins.2022.880178 (PMC9063408; doi:10.3389/fnins.2022.880178)
Supplement: Supplementary file 1 [file Data_Sheet_1.docx]

Supplementary Material

**Supplementary figures**

#
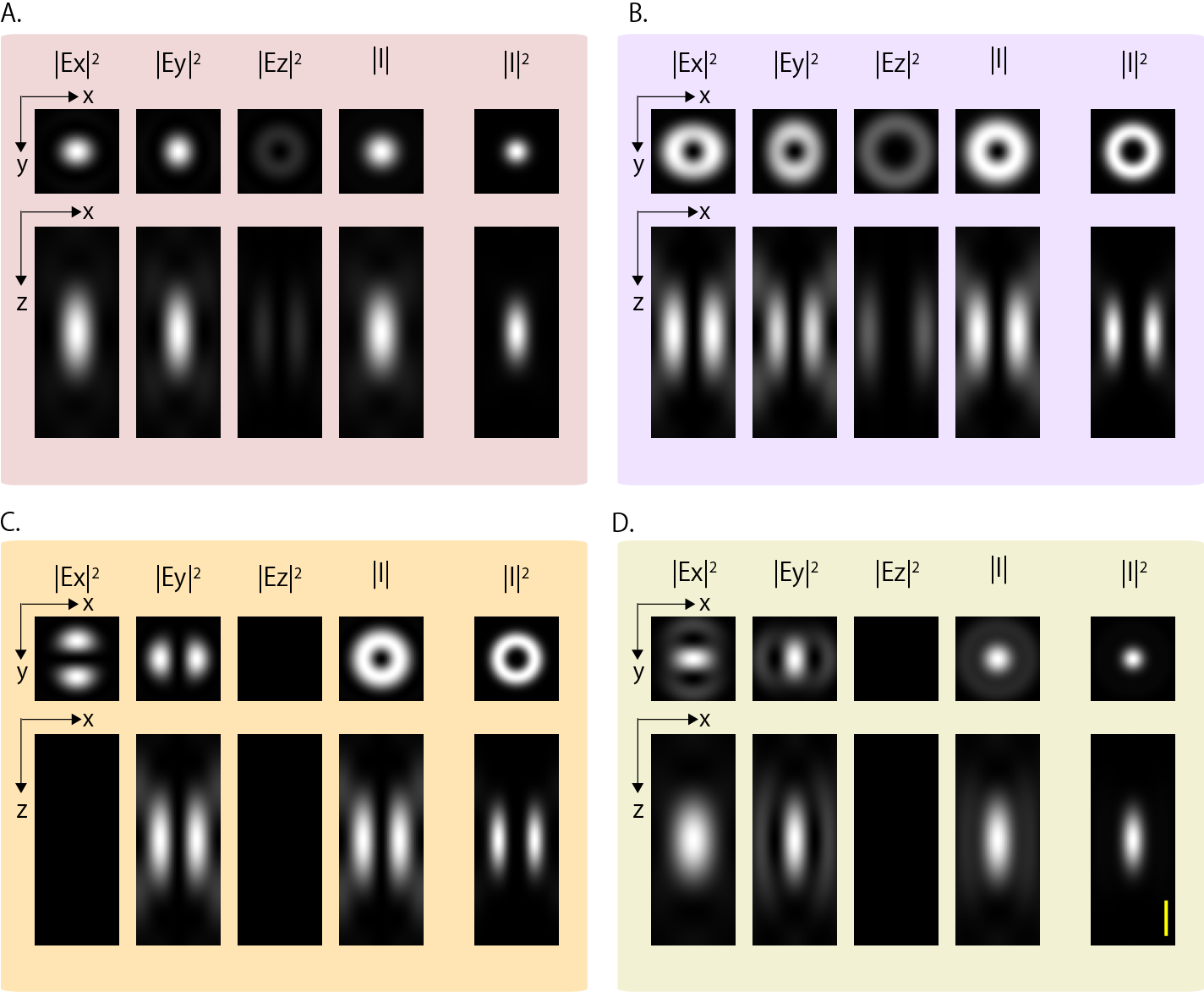


**Supplementary Figure 1.** Simulation results: Two-photon point spread function (PSF) obtained by polarization and phase modulation. x-z images obtained using (A) circularly polarized, (B) circularly polarized vortex, (C) azimuthally polarized, and (D) azimuthally polarized vortex beams as the excitation beams. The silicone immersion objective lens with NA = 1.3 was used to focus these excitation beams. $\left| I \right|=\left| E_{x} \right|^{2}+\left| E_{y} \right|^{2}+\left| E_{z} \right|^{2}$. Scale bar: 500 nm.


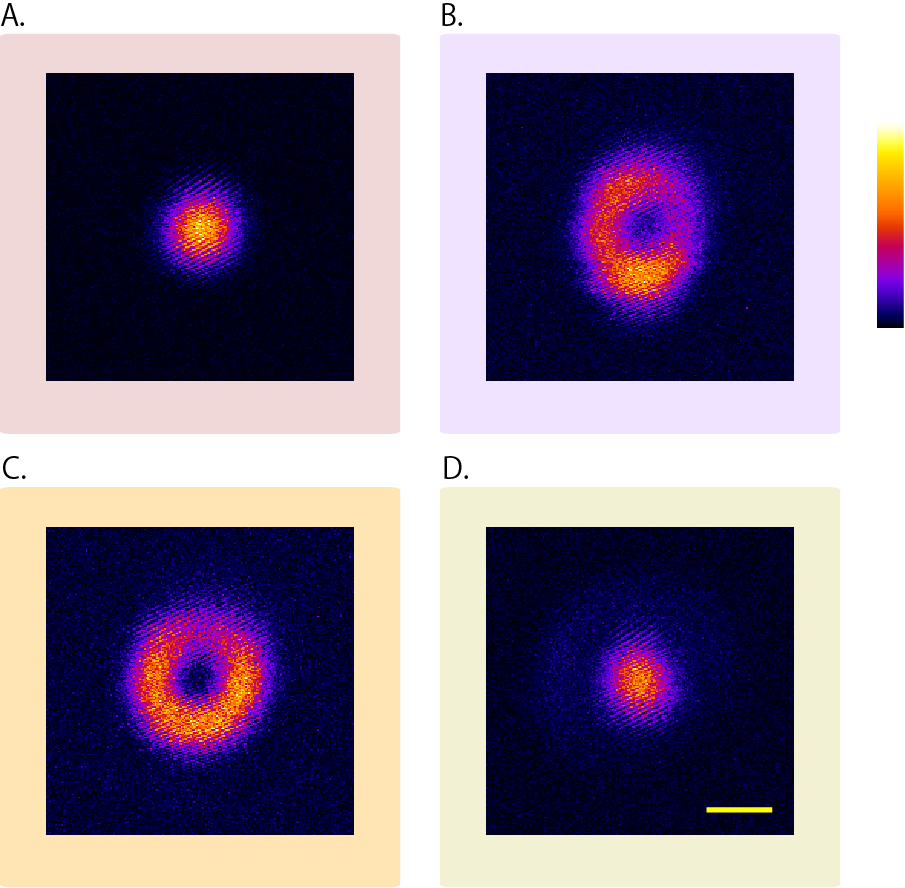


**Supplementary Figure 2.** Experimental results: The x-y images of the observed 0.2-µm-diameter fluorescent bead obtained using (A) circularly polarized, (B) linearly polarized vortex, (C) azimuthally polarized, and (D) azimuthally polarized vortex beams as the excitation beams. These beams were focused using a silicone immersion objective lens with NA = 1.3. Scale bar: 1 µm.


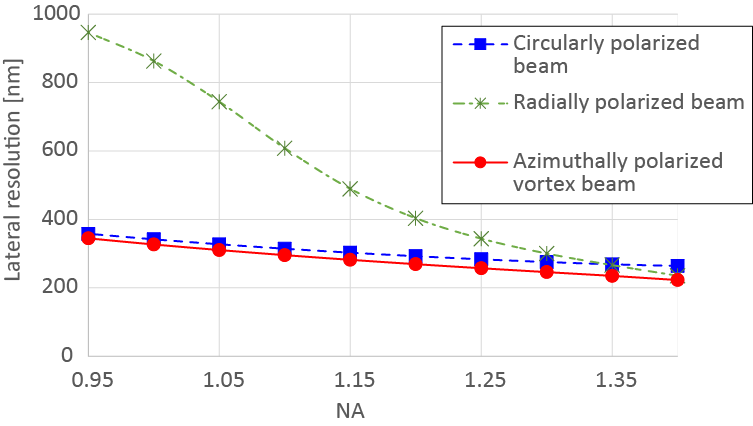


**Supplementary Figure 3.** Simulation results: Lateral resolutions modulated by the polarization- and phase-distribution of the excitation beams. The silicone immersion objective lens with NA = 1.3 was used to focus the excitation beams. The lateral resolution is the highest when the azimuthally polarized vortex beam is used.


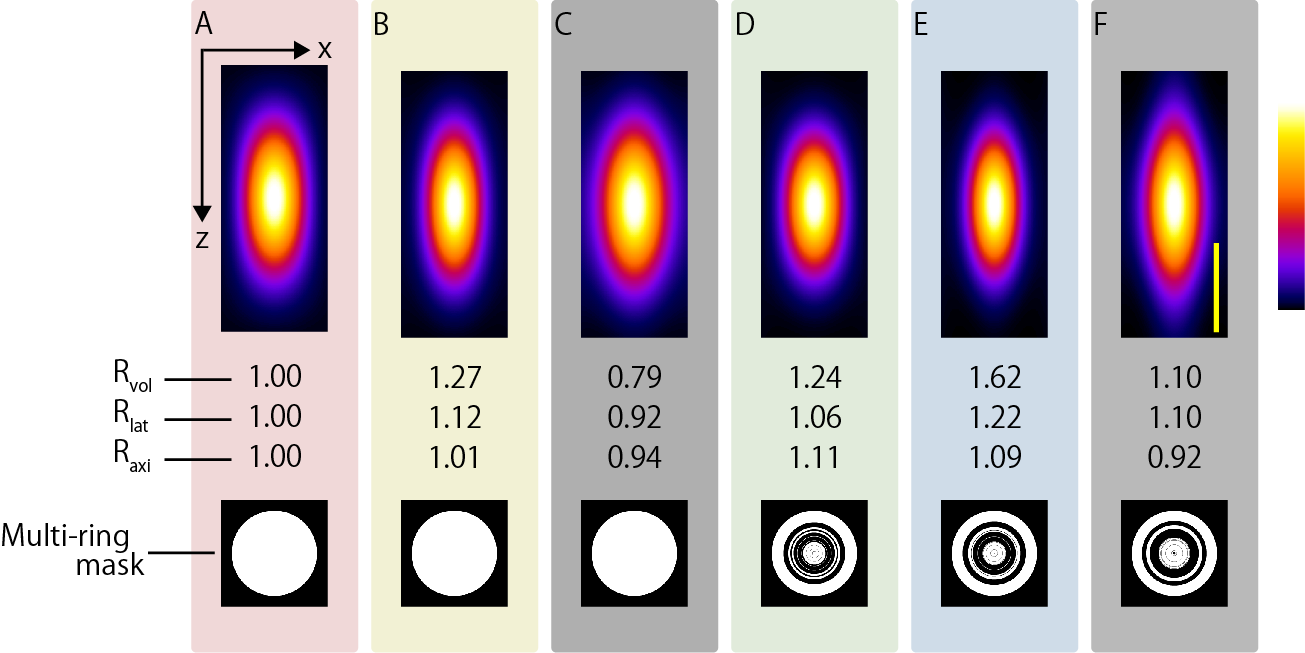


**Supplementary Figure 4.** Simulation results of two-photon PSF. Top: The x-z images were obtained using the (A) circularly polarized beam, (B) azimuthally polarized vortex beam, (C) radially polarized beam, (D) amplitude-distribution modulated circularly polarized beam, (E) amplitude-distribution modulated azimuthally polarized vortex beam, and (F) amplitude-distribution modulated radially polarized beam as the excitation beam. Bottom: Eight-ring mask pattern for realizing the second resolution-enhancement method. White and black regions represent the transmission and light-shielding areas, respectively. The beams were focused using a silicone immersion objective lens with NA = 1.3. Scale bar: 500 nm.


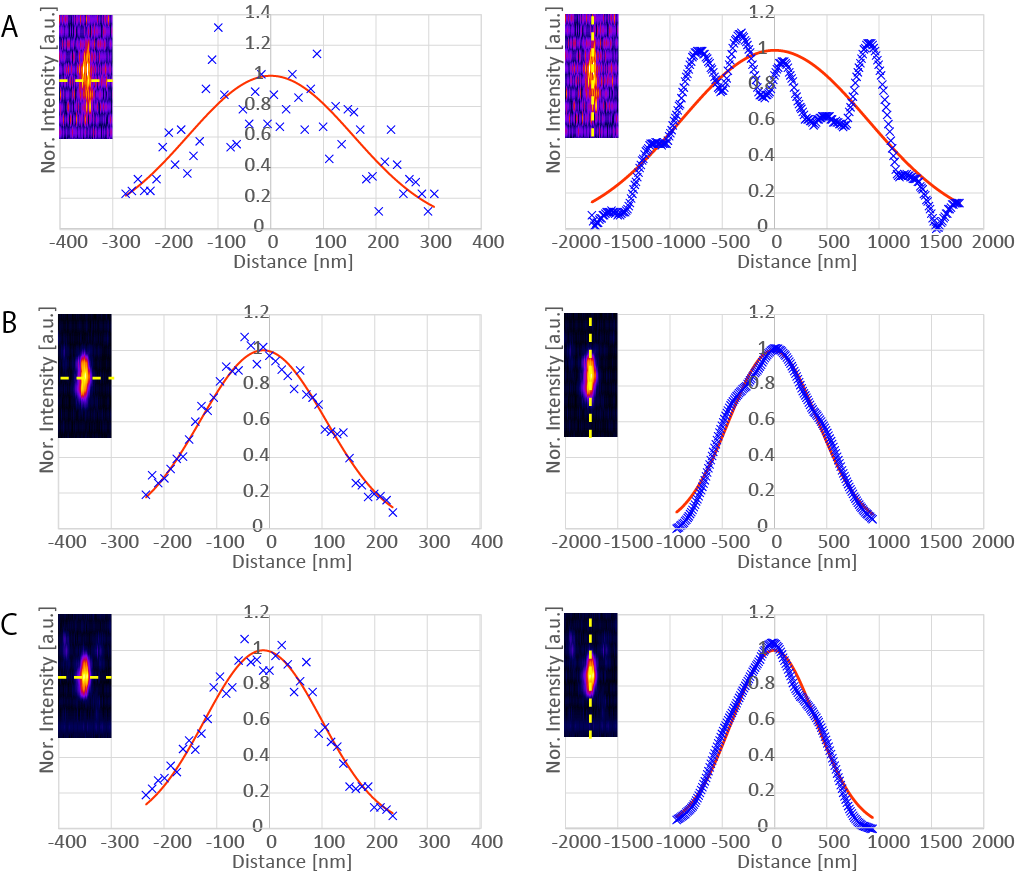


**Supplementary Figure 5.** Line profile and Gaussian fitting results of Figure 5. (A) Azimuthally polarized vortex excitation beam without SA correction. (B) Azimuthally polarized vortex excitation beam with SA correction. (C) Amplitude-distribution modulation azimuthally polarized vortex excitation beam with SA correction.


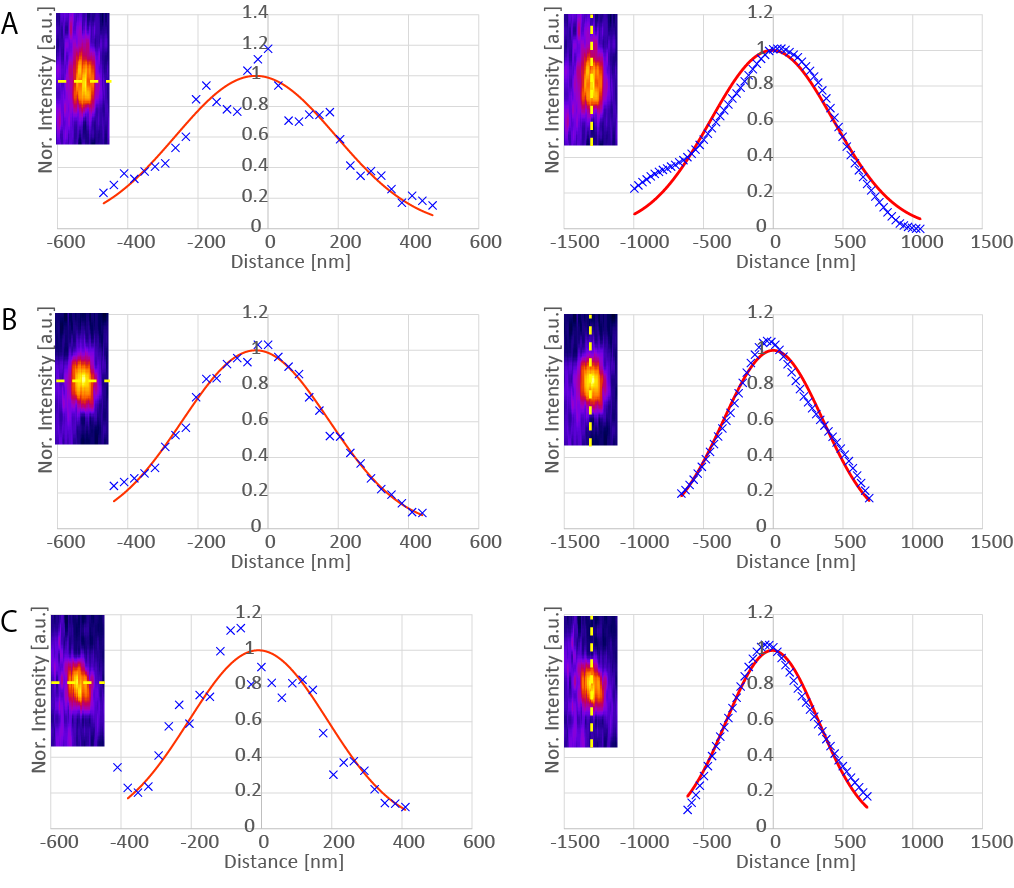


**Supplementary Figure 6.** Line profile and Gaussian fitting results of Figure 6. (A) Azimuthally polarized vortex excitation beam without SA correction. (B) Azimuthally polarized vortex excitation beam with SA correction. (C) Amplitude-distribution modulation azimuthally polarized vortex excitation beam with SA correction.

**Supplementary data**

**Algorithm for the multi-ring mask design**

Amplitude-modulation-type multi-ring masks for CP and APV excitation beams were designed using the following algorithm. This algorithm tolerates the generation of some sidelobes attributed to the mask, and it designs a mask pattern with the smallest PSF volume within the acceptable range of the sidelobe. Further, we added a method to search for mask patterns less affected by the aberration because aberration-induced sidelobes also occur if the correction is not performed completely. The PSF volumes were calculated using the PSF calculation equation (Ohtake et al., 2008), and the residual aberration wavefronts were calculated from the spherical aberration calculation (Matsumoto et al., 2015). For the sake of simplicity, we explain how to design a double-ring mask pattern; the eight-ring mask used in this report can be easily designed by adding position parameters and For loops to this algorithm.

**Algorithm** Multi-ring mask design

**Inputs:** *NA*: numerical aperture of the objective lens, *n*: refractive index of immersion fluid,

*λ*: wavelength of the excitation beam, *A*: Amplitude distribution of the excitation beam, $\emptyset_{rs}$phi_rs: wavefront of the residual spherical aberration.

**Procedure** SEARCH_MASK_POSITION (*NA*, *n*, *λ*, *A,* $\emptyset_{rs}$)

**int** *hh* = 0;

**double** $r_{1\_in}$*,* $r_{1\_out}$*,* $r_{2\_in}$*,* $r_{2\_out}$, $V_{min}$;

//$r_{1\_in}$, $r_{1\_out}$, $r_{2\_in}$, $r_{2\_out}$ are the ring positions

**while** *hh* = 0 **do**

*V*, $r_{1\_in\_tmp}$, $r_{1\_out\_tmp}$, $r_{2\_in\_tmp}$, $r_{2\_out\_tmp}$ $\leftarrow$ EVALUATING_AND_UPDATING_THE_MASK_POSITION(*NA*, *n*, *λ*, *A,* $r_{1\_in}$, $r_{1\_out}$, $r_{2\_in}$, $r_{2\_out}$, $\emptyset_{rs}$)

**if** (*V* = $V_{min}$) **then** *hh* $\leftarrow$ 1; **end if**

**if** (*V* < $V_{min}$) **then**

$V_{min}$ $\leftarrow$*V*;

$r_{1\_in}$ $\leftarrow$ $r_{1\_in\_tmp}$;

$r_{1\_out}$ $\leftarrow$ $r_{1\_out\_tmp}$;

$r_{2\_in}$ $\leftarrow$ $r_{2\_in\_tmp}$;

$r_{2\_out}$ $\leftarrow$ $r_{2\_out\_tmp}$;

**end if**  //Updating the position of the ring mask

**end while**

**return** $r_{1\_in}$, $r_{1\_out}$, $r_{2\_in}$, $r_{2\_out}$

**end procedure**

**Procedure** EVALUATING_AND_UPDATING_THE_MASK_POSITION (*NA*, *n*, *λ, A*, $r_{1\_in\_c}$, $r_{1\_out\_c}$, $r_{2\_in\_c}$, $r_{2\_out\_c}$, $\emptyset_{rs}$)

**double** *rstep*, $V_{min2}$, $s_{1\_max}$, $s_{2\_max}$;

**for** *ii* = -1 to 1 **do**

$r_{1\_in\_v}$ $\leftarrow$ $r_{1\_in\_c}$ + *rstep* × *ii*

**for** *jj* = -1 to 1 **do**

$r_{1\_out\_v}$ $\leftarrow$ $r_{1\_out\_c}$ + *rstep* × *jj*

**for** *kk* = -1 to 1 **do**

$r_{2\_in\_v}$ $\leftarrow$ $r_{2\_in\_c}$ + *rstep* × *kk*

**for** *ll*= -1 to 1 **do**

$r_{2\_out\_v}$ $\leftarrow$ $r_{2\_out\_c}$ + *rstep* × *ll*

*V*, $s_{1}$ $\leftarrow$ PSFCALCULATION (*NA*, *n*, *λ*, *A,* $r_{1\_in\_v}$, $r_{1\_out\_v}$, $r_{2\_in\_v}$, $r_{2\_out\_v}$)

//Calculating the PSF volume and sidelobe

$s_{2}\leftarrow$ SIDELOBE_SPH_CALCULATION (*NA*, *n*, *λ*, *A,* $r_{1\_in\_v}$, $r_{1\_out\_v}$, $r_{2\_in\_v}$, $r_{2\_out\_v}$, $\emptyset_{rs}$)

//Calculating the aberration-induced sidelobe

**if** (*V* < $V_{min2}$) **then**

**if** ($s_{1}$ < $s_{1\_max}$) **then**

**if** ($s_{2}$ < $s_{2\_max}$) **then**

$V_{min2}$ $\leftarrow$ *V*;

$r_{1\_in\_nc}$ $\leftarrow$ $r_{1\_in\_v}$;

$r_{1\_out\_nc}$ $\leftarrow$ -$r_{1\_out\_v}$;

$r_{2\_in\_nc}$ $\leftarrow$ $r_{2\_in\_v}$;

$r_{2\_out\_nc}$ $\leftarrow$ $r_{2\_out\_v}$;

**end if**

**end if**

**end if**

**end for**

**end for**

**end for**

**end for**

**return** $r_{1\_in\_nc}$, $r_{1\_out\_nc}$, $r_{2\_in\_nc}$, $r_{2\_out\_nc}$

**end procedure**

**References**

Matsumoto, N., Inoue, T., Matsumoto, A., and Okazaki, S. (2015). Correction of depth-induced spherical aberration for deep observation using two-photon excitation fluorescence microscopy with spatial light modulator. *Biomed. Opt. Exp.* 6:7, 2575-2587. doi: 10.1364/BOE.6.002575

Ohtake, Y., Ando, T., Inoue, T., Matsumoto, N., and Toyoda, H. (2008). Sidelobe reduction of tightly focused radially higher-order Laguerre-Gaussian beams using annular masks. Opt. Lett. 33:6, 617-619. doi: 10.1364/ol.33.000617
